# Therapeutic Drug Monitoring of the Subcutaneous Formulations of Infliximab and Vedolizumab—Current Knowledge and Future Directions

**DOI:** 10.3390/jcm15030972

**Published:** 2026-01-25

**Authors:** Ben Massouridis, Miles P. Sparrow

**Affiliations:** 1 Department of Gastroenterology, Alfred Health, Melbourne, VIC 3004, Australia; 2 School of Translational Medicine, Monash University, Melbourne, VIC 3004, Australia

**Keywords:** therapeutic drug monitoring, subcutaneous, infliximab, vedolizumab

## Abstract

Therapeutic drug monitoring of the intravenous formulations of infliximab in particular, but also vedolizumab, has become an important means of optimising these agents to minimise primary and secondary loss of response. More recently subcutaneous formulations of both infliximab and vedolizumab have become widely available. These new molecules offer patients the convenience of self-administration, and also have pharmacokinetic benefits via maintaining high drug levels, reducing the risk of the development of immunogenicity. It took many years before recommended therapeutic target ranges for intravenous biologics were agreed on, and it is now clear that target levels for the subcutaneous formulations are different, and further research is required before optimal drug levels are confirmed. This narrative review summarises the current literature of therapeutic drug monitoring of subcutaneous infliximab and vedolizumab, acknowledging that this evidence base is presently incomplete. We also aim to provide clinicians with some practical recommendations for the use of TDM with these formulations in clinical practice today. In summary, we recommend performing TDM of the IV formulations prior to switching and then measuring drug levels at 8 weeks after switching to SC infliximab and at 16 weeks for SC vedolizumab. We suggest provisional target drug levels obtained from post hoc analyses of >14 μg/mL for SC infliximab and 25–35 μg/mL for SC vedolizumab. We recommend performing reactive TDM in cases of loss of response to SC therapy. In conclusion we offer suggested areas for future research.

## 1. Introduction

Inflammatory bowel disease (IBD), encompassing Crohn’s disease (CD) and ulcerative colitis (UC), remains a chronic and complex immune-mediated condition requiring long-term immunosuppressive therapy in the majority of patients. The evolution of biologic therapies over the past two decades has transformed the management of IBD, including the advent of anti-tumour necrosis factor (anti-TNF) agents and integrin antagonists. Therapeutic drug monitoring (TDM) has emerged as a pivotal management tool aimed at helping to achieve sustained clinical remission and endoscopic healing.

Numerous systematic reviews and meta-analyses have confirmed the utility of reactive TDM in improving outcomes and guiding dose optimisation of the intravenous (IV) formulations of infliximab and, to a lesser extent, vedolizumab [1,2,3]. Proactive TDM, although not supported by all prospective studies, has been associated with reduced immunogenicity, lower treatment failure rates, better clinical outcomes and less need for IBD-related surgery or hospitalisation [4]. Until recently, however, TDM guidelines have only been developed around IV drug administration routes.

The recent global availability of subcutaneous (SC) formulations of infliximab and vedolizumab offer a new era of convenience and independence for patients. These formulations allow at-home administration, maintain steady-state drug exposure, and may promote better treatment persistence [5,6,7]. The comparative effectiveness of SC versus IV vedolizumab and infliximab has also been confirmed in systematic reviews, with no significant difference in rates of clinical remission or endoscopic healing [8].

As of 2025, SC infliximab and vedolizumab have been approved across multiple jurisdictions, including the European Medicines Agency (EMA), Food and Drug Administration (FDA) in the United States, and Therapeutic Goods Administration (TGA) in Australia, with increasing uptake in clinical practice. The pharmacokinetic profiles of SC formulations differ substantially from their IV versions, raising questions about the utility and practice of TDM with these agents. Key gaps in current understanding include the absence of validated trough level thresholds for SC infliximab and vedolizumab, the potential impact of altered absorption kinetics on drug exposure and immunogenicity, and the lack of robust data to guide TDM-based dose adjustments in clinical practice. While early simulation and real-world studies suggest a role for TDM with the SC formulations prospective evidence remains limited.

This review aims to synthesise the current literature regarding therapeutic drug monitoring of subcutaneous infliximab and vedolizumab formulations in IBD. We explore the pharmacological rationale, clinical trial evidence, potential switching strategies, and practical challenges associated with TDM in this evolving treatment landscape, while highlighting areas for future research.

## 2. Background and Rationale for TDM with SC Biologics

### 2.1. Therapeutic Drug Monitoring in IBD: Current and Evolving Practice

Therapeutic drug monitoring (TDM) refers to the measurement and interpretation of serum drug concentrations to inform treatment decisions, particularly dosing. In IBD, TDM has become an important component of personalised biologic dosing, especially for IV infliximab. Traditionally, TDM has been used reactively during loss of response or suspected immunogenicity, but a shift toward proactive drug level measurement, although not supported by all prospective studies, has demonstrated potential in improving long-term outcomes, including endoscopic healing, sustained remission, and reduced hospitalisations [4,9].

The rationale for TDM is supported by pharmacokinetic (PK) and pharmacodynamic (PD) principles: anti-TNF and anti-integrin agents display wide inter-individual variability in clearance rates, bioavailability, and immunogenicity. Subtherapeutic drug levels, often due to accelerated clearance or anti-drug antibodies (ADAs), have been associated with poor outcomes and treatment failure. Conversely, identifying optimal trough thresholds (e.g., ≥5 µg/mL for infliximab and ≥15 µg/mL for vedolizumab) has been associated with improved clinical efficacy [10,11].

### 2.2. Why Subcutaneous Formulations Have Changed the Landscape

Subcutaneous (SC) biologics such as infliximab (CT-P13 SC) and vedolizumab have recently become available, offering a more uniform PK profile and improved patient convenience. SC administration results in more stable serum concentrations compared to IV formulations, potentially reducing peak–trough fluctuations and the risk of immunogenicity associated with periods of low drug levels [5,6].

SC formulations also offer patient-centric advantages: self-administration reduces healthcare burden and may enhance treatment persistence, especially in stable patients. However, SC formulations also come with potential limitations. Differences in absorption kinetics, bioavailability, and injection-site variability could influence drug exposure, particularly in real-world populations. Additionally, validated TDM targets for SC formulations are not yet established, although it is recognised that these are different to target drug levels for IV formulations [12,13].

Theoretically, the stable and more predictable PK profile of SC biologics might reduce the need for frequent TDM. However, recent population PK simulation models and real-world cohorts suggest that TDM still plays a role particularly when switching from IV to SC formulations, managing loss of response, or investigating suspected immunogenicity [13,14].

## 3. Subcutaneous Infliximab (CT-P13 SC)

### 3.1. Pharmacokinetics and Clinical Evidence

CT-P13 SC is the subcutaneous formulation of the infliximab biosimilar CT-P13, designed to provide consistent drug exposure with enhanced patient convenience. Unlike intravenous (IV) infliximab, which produces high peaks and low troughs, SC infliximab generates a flatter concentration–time profile, theoretically minimising peak-related toxicity and trough-related loss of response and immunogenicity.

The pivotal phase I registration trial by Schreiber et al. demonstrated non-inferiority of SC CT-P13 compared to IV infliximab for the primary endpoint of week 22 trough concentrations in patients with active IBD. The geometric mean trough level at week 22 was approximately 21.45 µg/mL for SC (versus 2.93 µg/mL for IV), corresponding to a ratio of 1154% (90% CI, 786–1694%). The clinical remission rates at week 54 for UC and CD were also comparable between arms; 57.1% in CD and 68.4% in UC for the SC arm (versus 56% and 61.5% in the IV arms, respectively, *p* = NS) [5].

The LIBERTY studies, required for FDA registration of CT-P13 SC, comprised randomised, double-blind, placebo-controlled RCTs demonstrating significant superiority of subcutaneous infliximab over placebo in both maintenance-phase Crohn’s disease (CD) and ulcerative colitis (UC) patients. At Week 54, clinical remission rates were 62.3% vs. 32.1% (*p* < 0.0001) in CD and 43.2% vs. 20.8% (*p* < 0.0001) in UC. Additionally, endoscopic response in CD favoured SC therapy (51.1% vs. 17.9%; *p* < 0.0001). These results indicate that patients commencing IV infliximab may be transitioned to the SC formulation without loss of efficacy [15].

### 3.2. Immunogenicity and Monotherapy vs. Combination

Immunogenicity remains an important determinant of the efficacy of biologic therapies. In theory, SC infliximab’s stable PK profile with “high zone tolerance” and the absence of infusion-related troughs may reduce the risk of immunogenicity, although data remain mixed. In the pivotal registration study, the proportion of ADA-positive patients gradually increased in the CT-P13 SC arm, while it did not further increase in patients who switched from IV to SC after week 30. From weeks 38 to 54, the proportions of ADA-positive patients were not statistically different between the SC and IV arms (W38: *p* = 0.8603; W46: *p* = 0.1576; W54: *p* = 0.3787). Over the entire study (W0–W54), similar proportions of patients in each group converted to ADA-positive status (SC: 69.7%, 46/66 vs. IV: 63.5%, 40/63, *p* = NS). Importantly, a significantly smaller proportion of patients in the SC arm developed neutralising antibodies compared with the IV arm (18.2%, 12/66 vs. 36.9%, 24/65; *p* = 0.0194). Neutralising ADAs bind directly to functional regions of monoclonal antibodies, thereby blocking the drug’s pharmacological activity. In contrast, non-neutralising ADAs bind to parts of the monoclonal antibody that do not interfere with target binding. Although they still form immune complexes that increase drug clearance, their effects are less than neutralising ADAs. These findings suggest that while overall ADA development was comparable between routes, SC administration may reduce the risk of clinically relevant neutralising antibody formation [5].

Whether combination immunosuppression (e.g., azathioprine, methotrexate) is necessary alongside SC infliximab has been addressed in recent post hoc analyses. In the LIBERTY-CD and LIBERTY-UC studies, outcomes with SC infliximab were stratified by baseline immunosuppressant use. Clinical remission and endoscopic response rates at week 54 were comparable between patients receiving SC infliximab with or without concomitant immunosuppressants, suggesting that combination therapy did not significantly influence efficacy [16].

Similarly, an earlier pooled post hoc analysis of randomised trial data also found no significant advantage of combining SC infliximab with immunosuppressants over monotherapy. Clinical response, remission, and pharmacokinetic outcomes were maintained irrespective of immunosuppressant use [17].

These findings align with broader observations that immunogenicity risk in infliximab therapy may be influenced by route of administration, baseline disease activity, and prior immunogenic exposures, though definitive conclusions for SC infliximab are pending. Reviews and pharmacokinetic analyses suggest that therapeutic drug monitoring (TDM), particularly during and after switching, may provide earlier detection of immunogenicity and help identify patients at risk [9,18]. Together, these data suggest that while combination therapy may not confer additional benefit in terms of efficacy or drug exposure with SC infliximab, longer-term safety and immunogenicity surveillance will be important.

### 3.3. Therapeutic Drug Monitoring in SC Infliximab

While TDM with IV infliximab is well established, its role in SC CT-P13 is still evolving. A pharmacokinetic simulation study by Wang et al. demonstrated that following an IV-to-SC switch (per label), serum trough concentrations (Ctrough) gradually rise over approximately eight weeks before reaching steady state (SS), whereas vedolizumab requires around 16 weeks [1]. At steady state, the median simulated Ctrough for SC infliximab was 12.8 μg/mL (interquartile range [IQR]: 9.2–17.6 μg/mL), illustrating substantial drug exposure achieved with SC dosing [13].

Accordingly, TDM is only informative when performed at two months after the first SC dose and once steady state has been achieved. Testing prior to this window risks underestimating actual exposure. The simulation also established a strong linear relationship between IV and SC steady-state trough levels, enabling clinicians to predict SC exposure from prior IV Ctrough values (R^2^ ≈ 0.89) [13]. Notably, even with the more stable PK profile of SC infliximab, individual variability in drug clearance and exposure persists, supporting a selective TDM strategy—particularly in patients with prior loss of response, body weight extremes, or other risk factors for underexposure [13].

Other authors have also advocated for ongoing TDM during IV-to-SC transitions, particularly in patients with previously unstable drug levels, recent loss of response, or at high immunogenic risk [18]. The multicentre, retrospective TIME study evaluated real-world IV to SC switching for infliximab and vedolizumab with TDM embedded in clinical pathways. Clinical remission was maintained at high rates after switching. For SC infliximab at 3, 6, and 12 months, the clinical remission rates were 87%, 86%, and 63%, respectively [19]. Remission rates were higher in patients who were already in remission at the time of switch, supporting elective switch policies during stable disease. The study’s primary endpoint was 12-month remission stratified by switch timing; outcomes remained robust across the study period. These data demonstrate the feasibility of implementing TDM alongside structured switch protocols across multiple centres [19].

While Loftus et al. highlighted the absence of a defined therapeutic range for SC infliximab, recent real-world and modelling studies suggest distinct thresholds are emerging [12]. The REMSWITCH study demonstrated that switching from IV to SC infliximab increased trough levels significantly: from 9.8 ± 6.4 µg/mL to 14.4 ± 5.7 µg/mL (*p* < 0.0001). Relapse risk was significantly higher in patients whose trough levels remained stable or decreased post-switch (31.8%) compared to those with increased levels (7.1%, *p* = 0.024) [20].

In a multicentre cohort by Smith et al., elective IV-to-SC switching was undertaken in patients predominantly in remission. Remission was maintained in over 90% at 3, 6, and 12 months post-switch, with stable CRP, faecal calprotectin, and Ctrough levels throughout. Median infliximab levels increased from 8.9 µg/dL at baseline to 16.0 µg/dL at 3 months (*p* < 0.001) and remained stable to 12 months [21]. Moreover, population pharmacokinetic modelling by Hanzel et al. characterised SC CT-P13 PK determinants and confirmed that the PK profile differs from IV and new target ranges for therapeutic drug monitoring are required. Clinical covariates affecting drug clearance were body weight, immunogenicity, and serum albumin [22]. At present, data on drug levels and BMI and albumin are mixed. Regarding BMI and drug levels, data appear different from population-based PK studies and real-world studies. PK studies suggest different drug levels with patient weights of 50 kg, >70 kg, and >120 kg whereas real world studies show no differences [20,22,23]. Similarly, regarding albumin, Hanzel et al. found that lower serum albumin concentration increased subcutaneous infliximab clearance by approximately 30% (32 g/L compared to 44 g/L) [22]. This contrasts with Roblin et al. who showed no association between hypoalbuminaemia and subcutaneous infliximab levels [23]. Although data are inconclusive, BMI and albumin should be considered when assessing response and dosing of subcutaneous infliximab. Together, these data, albeit from post hoc analyses rather than prospective studies, suggest that sustained SC infliximab troughs >14 µg/mL may be optimal for maintaining clinical remission and preventing immunogenicity [20,21,22].

## 4. Subcutaneous Vedolizumab

### 4.1. Pharmacokinetics and Clinical Evidence

Vedolizumab, a monoclonal antibody targeting the α4β7 integrin, is a cornerstone therapy for moderate-to-severe IBD. The subcutaneous (SC) formulation extends treatment flexibility post intravenous (IV) induction.

The VISIBLE trials served as pivotal studies: VISIBLE 1 demonstrated non-inferiority of SC vedolizumab (108 mg every 2 weeks) to IV vedolizumab (300 mg every 8 weeks) for maintaining clinical remission in ulcerative colitis (UC) at week 52 [24], and VISIBLE 2 replicated these findings in Crohn’s disease (CD), albeit with slightly lower remission rates than UC [25]. Safety was consistent across routes, with injection-site reactions common but generally mild.

A post hoc analysis incorporating both GEMINI (IV) and VISIBLE (SC) cohorts characterised the comparable exposure-efficacy relationships across both administration routes in UC and CD. Modelled steady-state trough concentrations and average concentrations correlated similarly with week-52 clinical remission for both SC and IV vedolizumab, supporting interchangeable efficacy during maintenance [6].

Further analyses considered real-world dynamics: post hoc VISIBLE data revealed that transitions from IV to SC preserved therapeutic response in patients in remission, that dose escalation from biweekly to weekly dosing regained response in ≥45% of those with loss of response, and that restarting SC vedolizumab after treatment interruptions (1–46 weeks) maintained efficacy in most patients [26].

Long-term safety was established in the VISIBLE-OLE extension, where >90% of SC-injected patients self-administered their doses at home. Over extended follow-up, SC vedolizumab demonstrated sustained clinical remission and low immunogenicity rates. ADA positivity was notably lower among patients initially receiving SC or IV drug than those starting on placebo [27].

### 4.2. Exposure–Response Relationship, Including for Endoscopic Healing

Emerging pharmacokinetic–pharmacodynamic (PK–PD) modelling from post hoc analyses of the GEMINI studies has previously defined exposure–response relationships for IV vedolizumab, with distinctions between clinical remission and endoscopic healing [6]. For SC vedolizumab, in VISIBLE 1 the Week 52 endoscopic healing rates increased across trough concentration quartiles, rising from 50% in the lowest quartile to 89% in the highest quartile. Notably, similar exposure–response trends were seen for both SC and IV routes, reinforcing that higher serum levels are linked to improved endoscopic healing regardless of route [28].

Recent real-world studies provide encouraging insights into the durability of therapeutic response and pharmacokinetics when transitioning from intravenous (IV) to subcutaneous (SC) vedolizumab, supporting the initial RCT data. A large multicentre UK study by Lim et al., collected data from 563 patients (187 CD, 376 UC) and reported high treatment persistence (84% at 12 months), good rates of clinical and biochemical remission (68% and 54%, respectively), and low rates of immunogenicity (4% ADA, no neutralising antibodies) [29]. Similar outcomes have since also been reproduced in smaller cohorts. In a prospective cohort study by Wiken et al., 108 IBD patients (57 CD, 51 UC) switched from IV to SC vedolizumab. Drug persistence at 18 months was 73.6% (95% CI: 64.2–80.1%). Those already in clinical remission at the time of switch were significantly less likely to discontinue SC therapy (HR = 0.34; 95% CI: 0.16–0.73; *p* = 0.006). Although mean vedolizumab concentrations remained high (39.1 μg/mL among patients on ≥14-day dosing), the study did not demonstrate a clear association between serum drug levels and clinical outcomes; clinical remission and biochemical markers were largely maintained irrespective of concentration variability, and no therapeutic threshold predictive of response was identified [14].

A single-centre report from Fric et al. demonstrated that switching to SC vedolizumab (*n* = 24) significantly increased trough concentrations with mean serum levels rising from IV to SC (22.86 μg/mL to 35.62 μg/mL, *p* = 0.002). Despite higher exposure, higher trough levels of subcutaneous vedolizumab were not associated with improved clinical outcomes [30]. These post hoc analyses from RCTs and real-world data demonstrate emerging exposure–response trends with SC vedolizumab; however, validated, target therapeutic ranges remain to be confirmed [10].

### 4.3. Role of TDM in SC Vedolizumab

TDM of SC vedolizumab may be informative in patients switching from the IV formulation. A pharmacokinetic modelling study by Wang et al. demonstrated that after switching from IV to SC vedolizumab, steady state is not reached until approximately 16 weeks [13]. Before this time, trough levels are rising and do not accurately reflect long-term exposure, making early TDM less informative [13]. Importantly, the study showed a linear relationship between steady-state IV and SC trough concentrations, allowing clinicians to predict SC exposure from recent IV measurements prior to switching [13]. This provides a practical framework for using TDM to identify patients at risk of underexposure and guide appropriate monitoring strategies during the switch process. Despite the more stable concentration–time profile of SC vedolizumab, individual variability persists, supporting selective use of TDM in clinical practice.

## 5. Practical and Logistical Considerations with SC TDM of Infliximab and Vedolizumab

### Assay Availability and Validation

The use of TDM during the transition from intravenous (IV) to subcutaneous (SC) formulations of infliximab and vedolizumab requires an understanding of current assay methodologies and limitations. Most commercial TDM assays (e.g., drug-sensitive or drug-tolerant ELISAs, homogeneous mobility shift assays [HMSA], and rapid point-of-care tests such as Quantum Blue) were validated using datasets derived from patients treated with IV formulations. While these assays can accurately quantify SC drug levels, the higher steady-state concentrations achieved with SC therapy are not directly comparable to IV-derived therapeutic thresholds [9,12].

Emerging data now support distinct SC-specific therapeutic ranges, although ideally these require validation in prospective studies incorporating TDM. These data are summarised in Table 1.

- SC infliximab (CT-P13): Median steady-state troughs are consistently higher than IV, with pivotal data showing Week 22 geometric mean troughs of ~21 µg/mL (SC) vs. ~3 µg/mL (IV) [5]. Real-world data from the REMSWITCH study further suggest that maintaining troughs >14 µg/mL may protect against relapse [20].
- SC vedolizumab: Population PK modelling and post hoc exposure–response analyses from the GEMINI and VISIBLE cohorts indicate that Week 52 clinical remission and endoscopic healing are maximised with troughs in the 25–35 µg/mL range, with median SC steady-state troughs reported around 31.5 μg/mL (IQR 23.2–42.0) [13,25,27].

## 6. Evidence Gaps and Future Directions

Despite the promise of subcutaneous (SC) formulations of infliximab and vedolizumab, substantial gaps remain in our understanding of how best to implement therapeutic drug monitoring (TDM) in this setting. Tursi et al. found that switching rates from IV to SC vedolizumab were lower than expected in some real-world settings, potentially reflecting physician uncertainty around TDM applicability, patient hesitation, or delayed access to SC formulations [31].

### 6.1. Absence of SC-Specific Trough Targets

The most critical TDM-related limitation is the lack of validated therapeutic thresholds for SC formulations. Existing TDM frameworks rely heavily on exposure–response relationships derived from intravenous (IV) administration. For example, maintenance trough targets such as ≥5 µg/mL for infliximab or ≥15 µg/mL for vedolizumab have been proposed for IV regimens, but these targets are not applicable for SC dosing regimens [6,10].

### 6.2. Limited Prospective TDM Studies

Most available data on SC biologics comes from post hoc analyses of registration trials or simulation models, but prospective investigator-initiated studies are emerging. The ongoing DISCUS-IBD trial by Little et al. is designed to evaluate whether patients on dose-intensified IV infliximab can successfully de-escalate to SC therapy using TDM-informed strategies [32]. Approximately 120 patients are being recruited across 13 Australian sites. Participants are randomised either to continue their existing intensified IV regimen or to de-escalate to SC infliximab with stratified dosing according to prior IV dosing. TDM is performed every 12 weeks, enabling the correlation of drug exposure with risk of flare. In parallel, the SISS trial (Switching from Intravenous to Subcutaneous infliximab in IBD), coordinated by Chetwood et al., is another prospective study actively recruiting. Patients stable on standard-dosing IV infliximab will be randomised either to continue IV therapy or to switch to SC infliximab (120 mg every 2 weeks) and followed for 48 weeks, with analyses including TDM data from both IV and SC arms [33].

### 6.3. Immunogenicity and the Role of Combination Therapy

Data on immunogenicity with SC biologics and whether patients on SC formulations require concomitant immunomodulators are still evolving. In the CT-P13 SC registration programme, overall ADA conversion was similar between SC and IV arms from Week 0 to 54, but neutralising antibodies (NAbs) were significantly less frequent with SC dosing (18.2% [12/66] vs. 36.9% [24/65]; *p* = 0.0194), irrespective of whether patients were receiving monotherapy or immunomodulator combination therapy [5]. In the LIBERTY RCTs of CT-P13 SC, efficacy outcomes at Week 54 were superior to placebo in both CD and UC, and subgroup analyses did not show a consistent efficacy advantage for combination therapy over monotherapy, although these studies were not powered to show outcome differences between monotherapy and combination therapy patients [15,16,17].

For vedolizumab, immunogenicity rates remain low in both IV and SC registration studies, with no signal that SC therapy increases ADA risk. Long-term extension data report low ADA/NAb incidence and similar safety across routes, although these data are more limited when compared to SC infliximab [23,27]. Real-world cohorts of SC vedolizumab mirror this profile: a UK multicentre study reported ADA ~4% and no NAbs, alongside high 12-month persistence [29].

Together, these data suggest that while overall ADA risk is comparable between routes, NAb formation are lower with SC infliximab, and routine combination immunosuppression is not always required for SC formulations, though ongoing prospective studies are required to confirm these findings [6,16,24,27]. Future adequately powered studies should confirm whether the PK profile of SC formulations sufficiently mitigates ADA risk in monotherapy patients. Similarly, the role of the HLA-DQA1*05 variant in predicting immunogenicity to SC infliximab should be confirmed.

### 6.4. Point-of-Care and At-Home Monitoring

The convergence of SC delivery and remote TDM is conceptually attractive, aligning well with decentralised care models. Devices for capillary blood sampling, such as dried blood spots (DBS) and microsampling platforms, coupled with rapid point-of-care assays, could allow patients to self-monitor drug levels at home and share results remotely with providers via web-based portals. Although recent innovations in DBS and microsampling technology have sparked pilot studies, real-world implementation lags behind, and only a few assays are commercially available or validated for SC infliximab or vedolizumab. Importantly, the fingerPRICKS study by Chee et al. demonstrated the feasibility and acceptability of patient-led home-based DBS sampling for TDM in IBD, showing good concordance with venepuncture assays and high patient engagement [34]. These findings are consistent with expert opinion recommendations that point of care TDM is integrated into personalised treatment strategies to optimise biologic therapy [35]. Further studies evaluating the accuracy, patient usability, and clinical impact of home-based TDM for SC biologics are needed. Currently, variability in assay types (ELISA vs. HMSA vs. point-of-care) complicates cross-study comparison and limits generalisability of trough-level targets. Ideally, there is a need to harmonise assay platforms and reporting units across regions for both lab-based and point of care assays.

## 7. Conclusions and Clinical Recommendations

The emergence of SC formulations of infliximab and vedolizumab marks a significant advance in the therapeutic landscape for IBD. These agents offer increased convenience, and more stable pharmacokinetic profiles.

**Figure 1:**
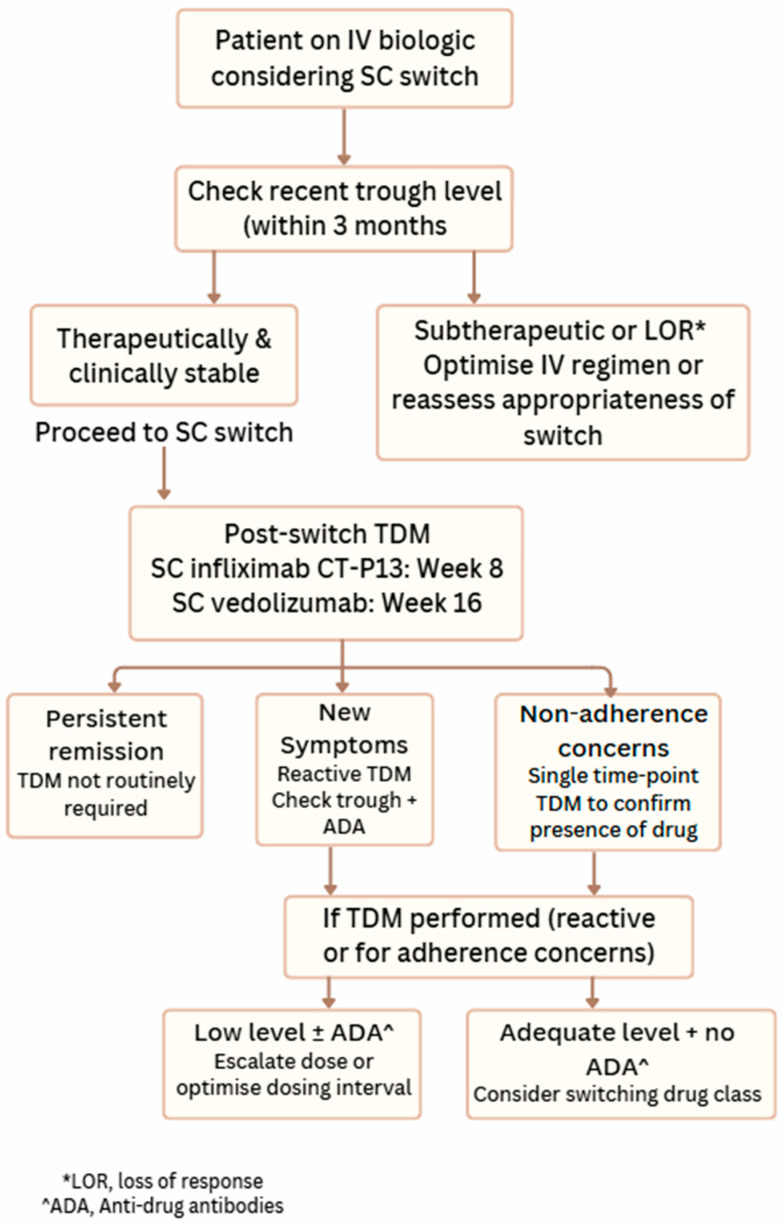
Suggested TDM algorithm for SC biologics.

Although TDM is well-established for intravenous biologics, its role in the SC era remains in evolution. Early data from SC registration trials suggest that TDM has clinical utility particularly when switching from IV to SC and managing suspected loss of response or investigating potential immunogenicity. However, the absence of validated SC-specific trough thresholds, limited assay calibration, and the lack of prospective TDM-driven studies highlight a need for caution and further research. Recommendations for the current use of TDM of SC infliximab and vedolizumab are given in Table 2 and Figure 1.

## Figures and Tables

**Table 1:** Suggested therapeutic ranges for SC infliximab and vedolizumab.

Drug (Formulation)	Reported Steady-State Troughs	Exposure-Response Observations	Suggested Provisional Maintenance Range
Infliximab (SC, CT-P13)	~21 µg/mL (W22, Schreiber et al.), REMSWITCH median 14–16 µg/mL	Relapse risk higher if trough <14 µg/mL	>14 µg/mL
Vedolizumab (SC)	Median 31.5 mg/L (IQR 23–42, Wang et al.);VISIBLE quartile analysis	Higher remission and healing with >25–30 µg/mL	25–35 µg/mL

**Table 2:** Recommendations for the current use of TDM of SC infliximab and vedolizumab.

Scenario	TDM Recommendation	Comments
Switching from IV to SC infliximab or vedolizumab	✔ Consider baseline TDM to confirm adequate exposure	Helps establish pre-switch pharmacokinetic status
Patient with suspected loss of response on SC	✔ Perform TDM to assess for low levels or ADAs	May support switch back to IV or dose escalation
Clinically stable patient on SC	✖ Routine TDM currently not recommended	No defined proactive thresholds yet available
Non-adherence concerns	✔ TDM can help assess drug presence or absence	Especially useful in self-administered SC setting

## Data Availability

No new data were created or analysed in this study.
